# Blood Pressure Trajectories for 16 Years and the Development of Left Ventricular Hypertrophy and Increased Left Atrial Size: The Korean Genome and Epidemiology Study

**DOI:** 10.1155/2022/6750317

**Published:** 2022-07-18

**Authors:** Seong Hwan Kim, Ju-Mi Lee, Seung Ku Lee, Chol Shin, Jae-Hyeong Park

**Affiliations:** ^1^Department of Cardiology, Institute of Human Genomic Study, Korea University Ansan Hospital, Ansan, Republic of Korea; ^2^Department of Preventive Medicine, Eulji College of Medicine, Daejeon, Republic of Korea; ^3^Institute of Human Genomic Study, Korea University Ansan Hospital, Ansan, Republic of Korea; ^4^Department of Cardiology in Internal Medicine, School of Medicine, Chungnam National University, Chungnam National University Hospital, Daejeon, Republic of Korea

## Abstract

**Background:**

Elevated single blood pressure (BP) measurement can be associated with the development of hypertension-mediated target organ damage including left ventricular hypertrophy (LVH) and left atrial (LA) enlargement (LAE). However, long-term patterns of BP and their effects on LVH and LAE are poorly understood. We evaluated the association between the BP trajectories and the presence of LVH and LAE.

**Methods:**

We analyzed a total of 2,565 participants (1,267 males, 47.8 ± 6.7 years old) from the first biennial examination (2001-2002) of the Korean Genome and Epidemiology Study. The presence of LVH and LAE was identified by echocardiography performed at the 8^th^ biennial examination (2015-2016). Latent mixture modeling was used to identify trajectories in mid-BP ((systolic BP + diastolic BP)/2) over time. Linear logistic regression was used for assessing BP trajectories with the outcomes.

**Results:**

We identified 4 distinct mid-BP trajectories: group 1 (lowest, 20.9%, *n* = 536), group 2 (36.2%, *n* = 928), group 3 (32.3%, *n* = 828), and group 4 (highest, 10.6%, *n* = 273). Compared with the lowest group, trajectories with elevated mid-BP had greater odds ratios having LVH and LAE by multivariable-adjusted regression models. Adjusted odd ratios for LVH were 2.033 (95% CI = 1.462–2.827, *P* < 0.001) for group 2, 3.446 (95% CI = 2.475–4.797, *P* < 0.001) for group 3, and 4.940 (95% CI = 3.318–7.356, *P* < 0.001) for group 4. Adjusted odd ratios for LAE were 1.200 (95% CI = 0.814–1.769, *P* = 0.358) for group 2, 1.599 (95% CI = 1.084–2.360, *P* = 0.018) for group 3, and 1.944 (95% CI = 1.212–3.118, *P* = 0.006) for group 4.

**Conclusions:**

Higher long-term mid-BP was an independent risk factor of cardiac structural changes such as LVH and LAE among middle-aged population.

## 1. Introduction

Hypertension is the most common and major cardiovascular risk factor. In hypertensive patients, hypertension-mediated target organ damage (HMOD) is associated with increased morbidity and mortality [1]. The identification of HMOD is pivotal in the initiation of antihypertensive treatment through risk stratification of hypertensive patients [1]. Left ventricular hypertrophy (LVH) and left atrial (LA) enlargement (LAE) are well-known cardiac phenotypes of HMOD. LVH comes from hypertrophy and remodeling of cardiomyocytes [2]. And, the presence of LVH associates with an increased risk of arrhythmias and sudden cardiac death [2]. Likewise, LAE and LA dysfunction are associated with an increased risk of atrial fibrillation and all-cause mortality [3, 4].

Single blood pressure (BP) levels or mean BP levels from short-term BP measurements for 24 hours in ambulatory BP monitoring are cross-sectionally associated with HMOD like LVH and LAE [5, 6]. BP changes over time, and patterns of BP change may differ among individuals. The evaluation of the effect of BP fluctuations over a long period on HMOD will provide significant evidence for long-term BP control. However, it has been studied insufficiently. Recently, trajectory analysis can evaluate the effect of long-term BP change and its relationship with a lifetime risk of cardiovascular disease [7]. Thus, we assessed the association with BP trajectories for 16 years and the presence of LVH and the LAE.

## 2. Methods

### 2.1. Study Cohort

This study was conducted with participants from a population-based cohort (Ansan cohort) within the Korean Genome Epidemiology Study (KoGES). This cohort is an ongoing longitudinal investigation funded by the Korean government (Korean National Research Institute of Health, Korean Centers for Disease Control and Prevention, and the Ministry of Health and Welfare) to investigate the genetic and environmental etiology of common metabolic and cardiovascular diseases in South Koreans [8, 9]. This cohort enrolled Koreans aged 40–69 years who resided in a city (Ansan-si, Gyeonggi-do, South Korea) without cardiovascular diseases between June 2001 and January 2003. Detailed information regarding study procedures is available in previous publications [8, 10].

### 2.2. Study Population

**Figure 1:**
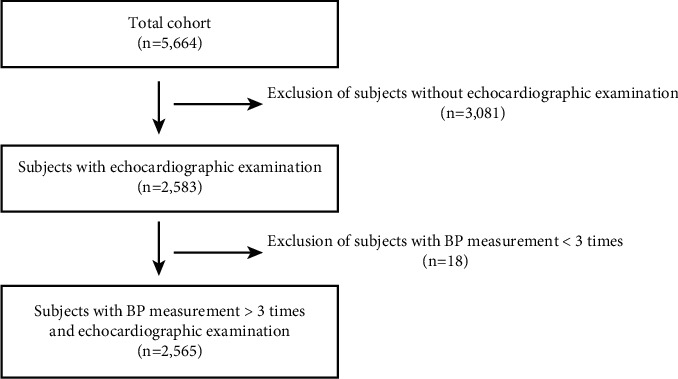
Study population flow diagram.

This analysis enrolled participants who attended a health examination from 2001 to 2016 (visits 1 to 8) in the Ansan cohort study, a part of the KoGES cohort study. We examined participants every two years. At every biennial visit, participants underwent pressure and pulse rate monitoring, body composition analysis, electrocardiography, pulmonary function test, chest X-ray, and blood chemistry test. Echocardiographic examination was included at the 4^th^ visit (from 2007 to 2008). Because echocardiographic examinations were an ancillary study of the original cohort, we excluded 3,081 participants without echocardiographic examinations to evaluate LVH and LAE at visit 8 (from 2015 to 2016) among the initial total of 5,664 participants. We additionally excluded 18 participants with BP measurements less than three times (Figure 1). Thus, we enrolled 2,565 participants (1,267 males, 47.8 ± 6.7 years old) in this study. All participants signed written informed consent forms, and the Institutional Review Board approved the study protocol. This study has been carried out following the latest version (2013) of the Code of Ethics of the World Medical Association (Declaration of Helsinki) for research involving humans.

### 2.3. Mid-BP Trajectories

We measured BP from the right brachial artery with a standard mercury sphygmomanometer (Baumanometer; WA Baum, NY, USA). According to the individual's arm circumference, resting BP was measured using an appropriate cuff size following the standardized protocol after at least 5 minutes of rest in the seated position using a standardized mercury sphygmomanometer. After the measurement of second and third BP values with at least 30 seconds intervals, we used the average value as systolic BP (SBP) and diastolic BP (DBP). Measurements were performed in a standardized way by trained researchers. The previous studies selected mid-BP ((SBP + DBP)/2) for identifying trajectories because mid-BP showed the greatest predictive utility for cardiovascular diseases compared with other single measures of BP (SBP, DBP, pulse pressure, or mean arterial pressure) [7, 11, 12]. Based on the previous study, we also used mid-BP to identify trajectories. The current sample has at least three times (minimum 3, maximum 8) of BP measurements over 16 years. We utilized all of this information to define trajectory groups. Following the previous study's steps [7], latent mixture modeling was used to identify trajectories in mid-BP over time. These models were fit using SAS PROC TRAJ [7, 13–15]. SAS PROC TRAJ fits longitudinal data as discrete mixture of two or more latent trajectories through maximum likelihood [7, 13–15]. It allows us to estimate the probabilities for multiple trajectories simultaneously instead of merely fitting the overall population mean [7]. We tested models with numbers and forms of potential trajectories. Model fit was selected using the Bayesian Information Criterion (BIC). We used a censored normal model appropriate for continuous outcomes. The scale for the time was the age at examination. Starting with all trajectory classes in a quadratic form, we examined models with five classes and then compared the BIC to that with 4, 3, 2, and 1, respectively. Once we had identified that the model with four classes fit best, we then compared the model fit of models with four classes with different functional forms. From this final model, we calculated the posterior predicted probability for each individual of being a member in each of the four classes. Participants clustered to the trajectory group for which they had the greatest posterior predictive probability. In our final model, participants were classified into trajectory groups with good discrimination [7].

### 2.4. Echocardiography and Definition of LVH and LAE

We performed conventional 2-dimensional echocardiography using commercially available echocardiographic machines (Vivid 7, GE Vingmed, Horten, Norway) according to the current recommendations of the American Society of Echocardiography and European Association of Cardiovascular Imaging [16]. Two-dimensional echocardiographic measurements included left ventricular (LV) end-diastolic dimension (LVEDD), LV end-systolic dimension (LVESV), interventricular septal thickness (IVST), and posterior wall thickness (LVPWT). The calculation of LV mass was done using a formula as follows: LV mass (g) = 0.8 × (1.04 × ((IVST + LVEDD + LVPWT) [3]—LVEDD [3])) + 0.6. LV mass was normalized for height (meter^2.7^), and LVH was defined as LV mass index >47 g/m^2.7^ in females and >50 g/m^2.7^ in males [17]. We calculated LA volumes with the modified Simpson's method on the apical 4 chamber and 2 chamber views. LA volume was indexed by body surface area and expressed as LA volume index (LAVI). WE defined LAE if LAVI is more than 34 mL/m^2^.

### 2.5. Confounders and Covariates

Detailed methods for the measurements used in the Ansan cohort study of the KoGES were previously reported elsewhere [18]. Trained research interviewers obtained participants' information, including personal medical history (hypertension, diabetic mellitus, and hypercholesterolemia), family history, and health behaviors (cigarette smoking, alcohol drinking, and exercise), using a standardized questionnaire.

We defined the presence of hypertension as SBP ≥140 mmHg and/or DBP ≥90 mmHg or taking antihypertensive medication on their questionnaires. If subjects have any one of the following, they were diagnosed with diabetes mellitus: (i) fasting plasma glucose ≥126 mg/dL; (ii) subjects who were using insulin or oral antidiabetic drugs. We used the duration of having diabetic mellitus. Hypercholesterolemia was diagnosed in subjects who had any one of the following: (i) total cholesterol ≥220 mg/dL; (ii) subjects who were using anti-hypercholesterolemia drugs or lifestyle modification for control of hypercholesterolemia. We used the duration of having hypercholesterolemia.

We assessed baseline cigarette smoking as three categories: never smoker, past smoker, and current smoker. Baseline alcohol drinking was classified into three categories: never drinker, past drinker, and current drinker. Baseline exercise was classified into two categories (yes or no): exercise more than 30 minutes at least two times for a week (yes) and others (no). Trained personnel measured the height and weight according to the written protocol. Body mass index (BMI) was calculated by weight (kg)/height (meter) [2]. Asian cutoff was used for evaluating obesity (BMI ≥25 kg/m^2^) and added all the years they were in the obese period.

Blood samples were collected after at least 8 hours of fasting to measure hemoglobin, blood glucose, total cholesterol, high-density lipoprotein cholesterol, triglyceride, creatinine, and high-sensitive C-reactive proteins.

### 2.6. Statistical Analysis

We used a latent mixture modeling to identify trajectories in mid-BP over time. Participants' characteristics according to the entire trajectory groups were expressed as mean values with standard deviation (or number and %). Participants' characteristics according to the total trajectory groups were expressed as mean values with standard deviation (or number and %). Analysis of Variance (ANOVA) test or chi-square test was used. The linear logistic regression assessed the associations of BP trajectories with the presence of LVH and the LAE. Odds ratio (OR) and 95% confidence interval (CI) were calculated. We performed all analyses with SAS software version 9.2 (SAS Institute, Cary, NC, USA).

## 3. Results

### 3.1. Participants' Characteristics according to Mid-BP Trajectory Groups

We analyzed a total of 2,565 participants (1,267 males, 47.8 ± 6.7 years old) and divided them into 4 groups according to mid-BP trajectory groups. Their baseline characteristics are shown in Table 1. The participants were 536 people (20.9%) for the 1^st^ group, 928 (36.2%) for the 2^nd^ group, 828 (32.3%) for the 3^rd^ group, and 273 (10.6%) for the 4^th^ group, respectively. The lowest mid-BP trajectory group had significantly higher proportion of female sex and lower proportion of cardiovascular risk factors including obesity, hypertension, diabetes, hypercholesterolemia, and current smoker. Also, the participants in the lowest mid-BP trajectory group were younger and had lower levels of SBP and DBP, total cholesterol level, and hemoglobin level. However, the frequency of exercise was similar among mid-BP trajectory groups.

### 3.2. Mid-BP Trajectories

**Figure 2:**
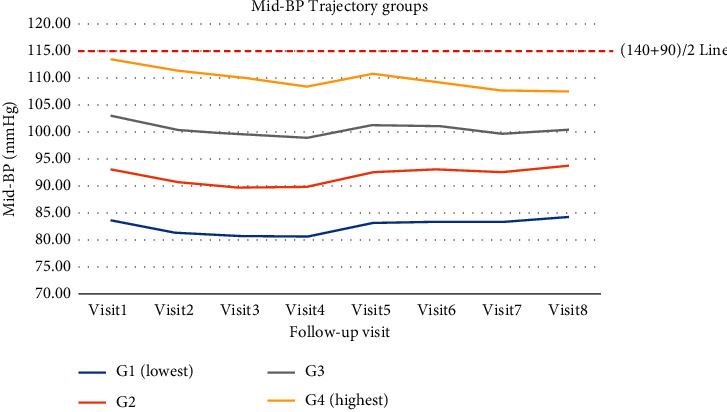
Mid-BP trajectory groups over time. These show mid-BP trajectories for 16 years (from visits 1 to 8) with mid-BP levels of each visit by trajectory groups. Group 1 is the lowest mid-BP group, and group 4 is the highest mid-BP group. Group 2 is the second-lowest mid-BP group and has a value lower than 95 mmHg. Group 3 is the third-lowest Mid-BP group and has a value lower than 103 mmHg.

We showed mid-BP trajectories for 16 years (from visits 1 to 8) with mid-BP levels of each visit by trajectory groups in Figure 2. Interestingly, in this population, the mid-BP trajectories were very stable for 16 years, which seems almost similar to the baseline level. Perhaps, this phenomenon was due to the middle-aged population after their BP was settled after early adulthood and well managed by cohort health evaluation. The baseline SBP levels of all groups were under 140 mmHg. The baseline levels of mid-BP were 83 mmHg (1^st^ group), 93 mmHg (2^nd^ group), 103 mmHg (3^rd^ group), and 113 mmHg (4^th^ group), respectively. Mid-BP levels of each visit by trajectory groups were lower (Supplementary Table 1). Group 1 is the lowest mid-BP group, and group 4 is the highest mid-BP group. Group 2 is the second-lowest mid-BP group and has a value lower than 95 mmHg. Group 3 is the third-lowest mid-BP group and has a value lower than 103 mmHg.

### 3.3. Mid-BP Trajectories and the Presence of LVH

The long-term mid-BP patterns of 16 years showed a significant positive relationship with the presence of LVH in both men and women (Table 2). Mid-BP levels more than 90 mmHg (2^nd^, 3^rd^, and 4^th^ groups) compared to under 90 mmHg (1^st^ group) had higher mid-BP values for a long time, and these groups had an increased risk for the presence of LVH. Before and after adjusting other variables, ORs for having the LVH showed a positive association in 2^nd^, 3^rd^, and 4^th^ groups and the trend of OR increased as in the trajectory groups. Compared to the female group, however, the association of the 2^nd^ group with the presence of LVH was not seen after the adjustment of other variables (Model II) in the male group (OR = 1.978, 95% CI = 0.996–3.925, *P*=0.051).

### 3.4. Mid-BP Trajectories and the Presence of LAE

Compared with the lowest mid-BP group for 16 years (visits 1 to 8), trajectories with elevated mid-BP groups (3^rd^ and 4^th^ groups) had greater odds ratios for having LAE (Table 3). Adjusted odd ratios for the LAE were 1.200 (95% CI = 0.814–1.769, *P*=0.358) for the 2^nd^ group, 1.599 (95% CI = 1.084–2.360, *P*=0.018) for the 3^rd^ group, and 1.944 (95% CI = 1.212–3.118, *P*=0.006) for the 4^th^ group in the total population. In women, unadjusted ORs for LAE were 1.622 (95% CI = 1.029–2.558, *P*=0.037) for the 2^nd^ group, 2.992 (95% CI = 1.918–4.667, *P* < 0.001) for the 3^rd^ group, and 3.879 (95% CI = 2.140–7.033, *P* < 0.001) for the 4^th^ group. After the adjustment of confounders and covariates, the significant association of mid-BP level with LAE was observed in the 3^rd^ (OR = 2.005, 95% CI = 1.249–3.218, *P*=0.004) and the 4^th^ (OR = 2.732, 95% CI = 1.455–5.128, *P*=0.002) groups. On the other hand, we could not find a statistically significant association among men.

## 4. Discussion

In this study, our results clearly showed that 16 years of long-term higher mid-BP trajectory groups were positively associated with the future presence of LVH in middle-aged males and females. Also, higher mid-BP trajectory groups were associated with future LAE in middle-aged females.

LVH and LAE are an important cardiac component of HMOD, and HMOD is associated with an increased risk of cardiovascular disease in patients with hypertension [19]. Because LVH comes from a maladaptive response to chronic overload of LV afterload, the antihypertensive medications which reduce LV afterload can reverse LVH [20]. Thus, identifying high-risk subjects developing HMOD can give us a chance to prevent future cardiovascular events. However, current risk prediction models include BP levels only at one time of the risk prediction and neglect the effect of BP levels over time [7]. The BP trajectory model can describe the course of BP variables over time [20]. Also, SBP increases with aging, and the patterns of BP change with aging may differ among individuals [21, 22].

To overcome single BP measurement weakness, investigators used ambulatory BP monitoring and its relation between new-onset abnormal LV geometry. In these studies, baseline nocturnal SBP was the most potent BP variable related to LVH progression [23]. However, ambulatory BP monitoring is still insufficient for the identification of high-risk individuals. Thus, we used long-time BP trajectories to identify LVH in this population-based cohort study.

### 4.1. Mid-BP Trajectory and LVH

Increased BP can be associated with an increased risk of LVH. One retrospective cohort study showed that recently diagnosed essential hypertension was associated with LVH [24]. Also, prehypertension also had increased risk of LVH compared to normal BP in several cross-sectional studies [25–27].

Studies of the LVH with long-time BP changes were rare. In addition, longitudinal studies have been performed only in small numbers of subjects and often for relatively short periods in this subject. Moreover, as far as we knew, the association of longitudinal BP trajectory patterns and LVH were never studied before. Regardless, all these studies' consensus, that increased BP made LVH, are consistent with our results and concept. Because a trajectory represents the pattern of a measured variable over age or time, this analytic method is good for evaluating the change of BP overtime on the new onset LVH in our population-based cohort study [28]. Researchers validated the utility of trajectory analysis of BP on cardiovascular disease in several cohort studies. Smitson et al. reported that the patients with increased SBP and DBP had an increased risk of heart failure and cardiovascular mortality of the aged population in the Cardiovascular Health Study [29]. Because the mid-BP showed the highest power in predicting cardiovascular disease than other BP measurements [30], we used mid-BP trajectories and demonstrated that the increased mid-BP trajectories were significantly associated with LVH and LAE. In our study, the group with slightly increased mid-BP (group 2) also had an increased risk of LVH (OR = 2.033, 95% CI = 1.462–2.827, *P* < 0.001). This result is consistent with previous studies showing that prehypertension can be associated with an increased risk of LVH [25, 31, 32]. General population with prehypertension had an increased risk of LVH than the normal BP group (OR = 2.10, 95% CI = 1.63–2.70) in 52,111 normal participants after adjustment of other variables [31]. The high-normal BP group was associated with an increased risk of LVH (23.2% vs. 9.0%) than the normal BP group in cross-sectional and longitudinal data including 1,397 normal populations [25]. In one meta-analysis including a total of 60,949 participants [32], subjects with prehypertension had a higher risk of LVH (concentric remodeling (OR = 1.89, 95% CI = 1.70–2.10, *P* < 0.001), eccentric LVH (OR = 1.65, 95% CI = 1.40–1.90, *P* < 0.001), and concentric LVH (OR = 2.09, 95% CI = 1.50–3.00, *P* < 0.001)).

LVH can be associated with other risk factors in addition to BP. BMI and obesity were the significant determinants of LVH in previous studies. Lieb et al. reported that increased BMI was an independent predictor of new-onset LVH in hypertensive patients with normal LV mass [33]. Bombelli et al. showed that obese individuals had three times increased risk of developing LVH than lean subjects and obesity was an essential factor for the new-onset LVH in diabetic patients [34]. We excluded the effect of BMI by duration of obesity in this analysis and found that the increased mid-BP trajectory group had a significantly increased risk of LVH. Along with BP levels, seven variables (age, smoking, BMI, office SBP and DBP, Cornell voltage on electrocardiography, and chronic kidney disease) were associated with LVH assessed by echocardiography [35]. Mancusi et al. reported that a scoring system from these seven variables can be used to predict LVH at echocardiographic examinations in low-risk hypertensive patients. This score can help clinicians in risk profiling and decision-making for untreated hypertensive patients [35].

Interestingly in this population, mid-BP trajectories were very stable for 16 years, which seems almost similar to the baseline level. Perhaps, this phenomenon may come from that the middle-aged population after their BP was settled after early adulthood and well managed by cohort health evaluation. Our study baseline SBP was all under 140 mmHg (Table 1). Moreover, all groups mid-BP were under the (140 + 90)/2 line (Figure 2). Because our study population's BP levels were very similar to the baseline levels, our study showed a long-term, slightly below hypertension level of SBP associated with the increased risk of LVH. Our results show that long-time high BP within the non-hypertension range can affect the HMOD because hypertension is not a cutoff type disease but a continuous spectrum of high BP depredating.

### 4.2. Mid-BP Trajectory and LAE

LAE (LAVI >34 mL/m^2^) can be associated with arterial hypertension, ischemic heart disease, heart failure, and mitral valvular disease [36, 37], and it is associated with an increased risk of atrial fibrillation and subsequent stroke [38]. Also, the use of antihypertensive medications can be associated with reverse remodeling of LA (decrease of LAVI) in hypertensive patients [39] and patients with isolated diastolic dysfunction [40]. Thus, early detection of LAE and early treatment are the best way to reduce future cardiovascular disease. In our study, increased mid-BP trajectories were associated with the LAE, especially in females. There was sex difference in the effect of mid-BP trajectories on the LAE. Although we have clear explanation of this sex difference, the incidence of LAE and hormonal effects can be possible explanations.

Our study has several strengths. First, our study showed clear insight that higher long-term (16 years) mid-BP groups were a risk factor for developing future LVH with a well-constructed community cohort. Second, we had the strength of having relatively large-scale research subjects (*n* = 2,565). Third, our study design had advantages such as not interrupting recall bias and selective reporting reduction on BP. Fourth, the final model considered AHA's simple seven healthy behaviors by adjusting age, sex, duration of obesity, duration of diabetic mellitus, duration of hypercholesterolemia, cigarette smoking, alcohol drinking, and exercise.

### 4.3. Limitations

Although this study evaluated long-term BP patterns, this study had several limitations. First, this study is a community cohort including only Koreans. Therefore, external validity may have limitations. Thus, the mid-BP trajectory groups identified may not be generalized to other population groups. External validity can be supported by future studies. Second, due to data limitations, our population's mid-BP trajectory patterns were monotonous. They maintain BP levels almost similar to the baseline level. Third, we could not analyze lifestyle changes such as cigarette smoking, alcohol drinking, and exercise routines during the follow-up period. Fourth, we did not check the prevalence of LVH or LAE at the baseline, and there could be a possibility of including participants with LVH or LAE at the baseline. However, the prevalence of LVH or LAE might be minimal because we included subjects without any cardiovascular disease at the baseline.

## 5. Conclusions

Although single BP measurement value is a well-known risk factor for LVH and LAE, our study suggests that long-term higher mid-BP was an independent risk factor for having LVH among middle-aged males and females. Also, higher mid-BP was an independent risk factor for having future LAE in middle-aged females. Because early identification of HMOD can reduce future cardiovascular disease in these patients, our mid-BP trajectories may evaluate high-risk individuals in the smart electronic health records era. This study suggested meaningful insight because it is consistent with other studies' concepts: well-performed small cohort study results and case-controlled study results. External validity can be supported with further studies such as national-wide retrospective cohort like the health insurance cohort.

## 6. Disclosure

All authors have completed the ICMJE uniform disclosure form. A part of this study was presented as a poster abstract at the 2022 AHA EPI Lifestyle Conference, on March 2, Chicago, IL, USA. The funders had no role in study design, data collection and analysis, decision to publish, or preparation of the manuscript.

## Figures and Tables

**Table 1:** Participants' characteristics according to mid-BP trajectory groups (trajectory groups defined by total).

	1^st^ group (lowest)	2^nd^ group	3^rd^ group	4^th^ group (highest)	*P* value
Participants (%)	536 (20.9%)	928 (36.2%)	828 (32.3%)	273 (10.6%)	<0.001
Female sex (%)	399 (74.4%)	460 (49.6%)	348 (42.0%)	91 (33.3%)	<0.001
Age (years)	45.8 ± 5.4	47.7 ± 6.7	48.9 ± 6.9	49.1 ± 7.1	<0.001
BMI (kg/m^2^)	23.4 ± 2.6	24.6 ± 2.8	25.5 ± 2.9	25.3 ± 2.7	<0.001
Obesity baseline (%)	125 (23.3%)	408 (44.0%)	441(53.3%)	147(53.9%)	<0.001
Duration of obesity (years)	1.8 ± 2.8	3.1 ± 3.2	3.8 ± 3.3	3.7 ± 3.3	<0.001
SBP (baseline, mmHg)	100.0 ± 8.3	111.3 ± 10.5	123.1 ± 13.1	136.1 ± 15.3	<0.001
DBP (baseline, mmHg)	66.6 ± 6.4	74.9 ± 7.4	83.3 ± 9.0	91.6 ± 9.0	<0.001
Mid-BP (baseline, mmHg)	83.3 ± 6.7	93.1 ± 8.2	103.2 ± 10.3	113.9 ± 11.2	<0.001
Hypertension (%)	1 (0.2%)	30 (3.2%)	86 (10.4%)	43 (15.8%)	<0.001
DM baseline (%)	17 (3.2%)	45 (4.9%)	46 (5.6%)	16 (5.9%)	0.188
Duration of DM (years)	0.4 ± 1.4	0.7 ± 1.9	0.9 ± 2.1	0.8 ± 1.9	<0.001
Hypercholesterolemia (baseline, %)	29 (5.4%)	108 (11.6%)	101 (12.2%)	40 (14.7%)	<0.001
Duration of hypercholesterolemia (years)	0.8 ± 1.5	0.9 ± 1.4	0.8 ± 1.4	0.9 ± 1.6	0.661
Cigarette smoking					<0.001
Never smoker (%)	425 (79.3%)	532 (57.3%)	443 (53.5%)	131 (48.0%)	
Past smoker (%)	40 (7.5%)	189 (20.4%)	171 (20.7%)	83 (30.4%)	
Current smoker (%)	71 (13.3%)	207 (22.3%)	214 (25.9%)	59 (21.6%)	
Alcohol drinking					<0.001
Never drinker (%)	310 (57.8%)	401 (43.2%)	289 (34.9%)	91 (33.3%)	
Past drinker (%)	22 (4.1%)	48 (5.2%)	45 (5.4%)	17 (6.2%)	
Current drinker (%)	204 (38.1%)	479 (51.6%)	494 (59.7%)	165 (60.4%)	
Exercise					
Yes (%)	220 (41.0%)	352 (37.9%)	306 (37.0%)	94 (34.4%)	0.266
No (%)	316 (59.0%)	576 (62.1%)	522 (63.0%)	179 (65.6%)	
Baseline chemical profiles					
Fasting glucose (mg/dL)	83.0 ± 19.6	87.3 ± 18.5	89.9 ± 25.7	90.5 ± 23.9	<0.001
Total cholesterol (mg/dL)	185.6 ± 32.1	196.1 ± 35.3	198.2 ± 34.3	203.8 ± 35.4	<0.001
BUN (mg/dL)	13.7 ± 3.3	14.0 ± 3.3	14.5 ± 3.6	14.6 ± 3.6	<0.001
Creatinine (mg/dL)	0.8 ± 0.2	0.9 ± 0.2	0.9 ± 0.2	0.9 ± 0.2	<0.001
CRP (mg/L)	0.2 ± 0.3	0.2 ± 0.3	0.2 ± 0.4	0.2 ± 0.3	0.005
Hemoglobin (g/dL)	12.9 ± 1.5	13.7 ± 1.6	14.0 ± 1.6	14.3 ± 1.4	<0.001

Data were expressed as mean ± SD or number of people (%). BMI: body mass index; BP: blood pressure; BUN: blood urea nitrogen; CRP : C-reactive protein; DBP: diastolic blood pressure; DM: diabetes mellitus; SBP: systolic blood pressure.

**Table 2:** Association between mid-BP trajectories and the presence of left ventricular hypertrophy.

		Unadjusted			Model I			Model II		
		OR	95% CI	*P* value	OR	95% CI	*P* value	OR	95% CI	*P* value
Total (*n* = 2,565)	1^st^ group (lowest, *n* = 536)	Ref.	Ref.	Ref.	Ref.	Ref.	Ref.	Ref.	Ref.	Ref.
	2^nd^ group (*n* = 928)	2.749	2.036–3.713	<0.001	2.513	1.856–3.402	<0.001	2.033	1.462–2.827	<0.001
	3^rd^ group (*n* = 828)	5.266	3.913–7.087	<0.001	4.630	3.429–6.252	<0.001	3.446	2.475–4.797	<0.001
	4^th^ group (highest, *n* = 273)	6.718	4.711–9.580	<0.001	5.891	4.114–8.436	<0.001	4.940	3.318–7.356	<0.001

Males (*n* = 1,267)	1^st^ group (lowest, *n* = 137)	Ref.	Ref.	Ref.	Ref.	Ref.	Ref.	Ref.	Ref.	Ref.
	2^nd^ group (*n* = 468)	2.980	1.547–5.742	0.001	2.905	1.505–5.607	0.002	1.978	0.996–3.925	0.051
	3^rd^ group (*n* = 480)	6.418	3.371–12.218	<0.001	6.204	3.254–11.829	<0.001	3.490	1.774–6.865	0.001
	4^th^ group (highest, *n* = 182)	8.182	4.130–16.211	<0.001	7.805	3.931–15.499	<0.001	4.573	2.220–9.422	<0.001

Females (*n* = 1,298)	1^st^ group (lowest, *n* = 399)	Ref.	Ref.	Ref.	Ref.	Ref.	Ref.	Ref.	Ref.	Ref.
	2^nd^ group (*n* = 460)	3.292	2.320–4.673	<0.001	2.906	2.037–4.145	<0.001	2.118	1.431–3.136	0.001
	3^rd^ group (*n* = 348)	6.299	4.397–9.022	<0.001	5.084	3.518–7.346	<0.001	3.793	2.530–5.684	<0.001
	4^th^ group (highest, *n* = 91)	9.305	5.596–15.471	<0.001	7.510	4.469–12.620	<0.001	7.367	4.146–13.092	<0.001

CI: confidence interval, OR: odd ratio; Ref.: reference. Model 1: adjusted for age. Model II: adjusted for sex, age, duration of obesity (years, Asian cutoff used, BMI ≥25 kg/m^2^), duration of diabetic mellitus (years, fasting glucose ≥126 mg/dL or medication), duration of hypercholesterolemia (years, total cholesterol ≥220 mg/dL), cigarette smoking (baseline 3 categories), alcohol drinking (baseline 3 categories), and exercise (baseline more than 30 min at least 2 times for a week, Y or N).

**Table 3:** Association between mid-BP trajectories and increased left atrial volume index (>34.0 mL/m^2^).

		Unadjusted			Model I			Model II		
		OR	95% CI	*P* value	OR	95% CI	*P* value	OR	95% CI	*P* value
Total (*n* = 2,565)	1^st^ group (lowest, *n* = 536)	Ref.	Ref.	Ref.	Ref.	Ref.	Ref.	Ref.	Ref.	Ref.
	2^nd^ group (*n* = 928)	1.427	0.986–2.064	0.059	1.350	0.924–1.972	0.122	1.200	0.814–1.769	0.358
	3^rd^ group (*n* = 828)	2.064	1.438–2.961	<0.001	1.882	1.291–2.743	0.001	1.599	1.084–2.360	0.018
	4^th^ group (highest, *n* = 273)	2.385	1.539–3.698	<0.001	2.221	1.403–3.516	<0.001	1.944	1.212–3.118	0.006

Males (*n* = 1,267)	1^st^ group (lowest, *n* = 137)	Ref.	Ref.	Ref.	Ref.	Ref.	Ref.	Ref.	Ref.	Ref.
	2^nd^ group (*n* = 468)	1.190	0.613–2.310	0.607	1.133	0.581–2.207	0.715	0.897	0.452–1.778	0.755
	3^rd^ group (*n* = 480)	1.403	0.730–2.698	0.310	1.307	0.677–2.523	0.425	0.924	0.464–1.838	0.822
	4^th^ group (highest, *n* = 182)	1.658	0.801–3.432	0.173	1.498	0.720–3.120	0.280	1.061	0.494–2.279	0.879

Females (*n* = 1,298)	1^st^ group (lowest, *n* = 399)	Ref.	Ref.	Ref.	Ref.	Ref.	Ref.	Ref.	Ref.	Ref.
	2^nd^ group (*n* = 460)	1.622	1.029–2.558	0.037	1.343	0.843–2.138	0.214	1.230	0.763–1.985	0.395
	3^rd^ group (*n* = 348)	2.992	1.918–4.667	<0.001	2.195	1.383–3.483	<0.001	2.005	1.249–3.218	0.004
	4^th^ group (highest, *n* = 91)	3.879	2.140–7.033	<0.001	2.818	1.524–5.209	0.001	2.732	1.455–5.128	0.002

CI: confidence interval, OR: odd ratio; Ref.: reference. Model I: adjusted for sex, and age. Model II: adjusted for sex, age, duration of obesity (years, Asian cut-off used, BMI ≥25 kg/m^2^), duration of diabetic mellitus (years, fasting glucose ≥126 mg/dL or medication), duration of hypercholesterolemia (years, total cholesterol ≥220 mg/dL), cigarette smoking (baseline 3 categories), alcohol drinking (baseline 3 categories), and exercise (baseline more than 30 min at least 2 times for a week, Y or N).

## Data Availability

The data supporting this research article are available from the corresponding author on reasonable request.

## Supplementary Materials

Supplementary Materials Supplementary Table 1: mid-BP levels (mmHg) of each visit by trajectory groups.
