# Supplementary material for: Blood Pressure Trajectories for 16 Years and the Development of Left Ventricular Hypertrophy and Increased Left Atrial Size: The Korean Genome and Epidemiology Study
Source: Int J Hypertens. 2022 Jul 18;2022:6750317. doi: 10.1155/2022/6750317 (PMC9313986; doi:10.1155/2022/6750317)
Supplement: Supplementary Materials — Supplementary Table 1: mid-BP levels (mmHg) of each visit by trajectory groups. [file 6750317.f1.docx]

**Supplementary Table 1.** Mid-BP levels (mmHg) of each visit by trajectory groups

|  | 1^st^ group (lowest)  (n=536) | 2^nd^ group  (n=928) | 3^rd^ group  (n=828) | 4^th^ group (highest)  (n=273) |
| --- | --- | --- | --- | --- |
| Visit 1 | 83.60 | 93.10 | 102.94 | 113.37 |
| Visit 2 | 81.35 | 90.78 | 100.44 | 111.39 |
| Visit 3 | 80.76 | 89.70 | 99.52 | 110.10 |
| Visit 4 | 80.67 | 89.87 | 98.97 | 108.43 |
| Visit 5 | 83.16 | 92.57 | 101.26 | 110.81 |
| Visit 6 | 83.39 | 93.10 | 101.09 | 109.33 |
| Visit 7 | 83.40 | 92.57 | 99.71 | 107.66 |
| Visit 8 | 84.23 | 93.73 | 100.46 | 107.49 |
